# Effective strategies to motivate nursing home residents in oral care and to prevent or reduce responsive behaviors to oral care: A systematic review

**DOI:** 10.1371/journal.pone.0178913

**Published:** 2017-06-13

**Authors:** Matthias Hoben, Angelle Kent, Nadia Kobagi, Kha Tu Huynh, Alix Clarke, Minn N. Yoon

**Affiliations:** 1 Faculty of Nursing, University of Alberta, Edmonton, Alberta, Canada; 2 School of Dentistry, Faculty of Medicine and Dentistry, University of Alberta, Edmonton, Alberta, Canada; TNO, NETHERLANDS

## Abstract

**Background:**

Poor oral health has been a persistent problem in nursing home residents for decades, with severe consequences for residents and the health care system. Two major barriers to providing appropriate oral care are residents’ responsive behaviors to oral care and residents’ lack of ability or motivation to perform oral care on their own.

**Objectives:**

To evaluate the effectiveness of strategies that nursing home care providers can apply to either prevent/overcome residents’ responsive behaviors to oral care, or enable/motivate residents to perform their own oral care.

**Materials and methods:**

We searched the databases Medline, EMBASE, Evidence Based Reviews–Cochrane Central Register of Controlled Trials, CINAHL, and Web of Science for intervention studies assessing the effectiveness of eligible strategies. Two reviewers independently (a) screened titles, abstracts and retrieved full-texts; (b) searched key journal contents, key author publications, and reference lists of all included studies; and (c) assessed methodological quality of included studies. Discrepancies at any stage were resolved by consensus. We conducted a narrative synthesis of study results.

**Results:**

We included three one-group pre-test, post-test studies, and one cross-sectional study. Methodological quality was low (n = 3) and low moderate (n = 1). Two studies assessed strategies to enable/motivate nursing home residents to perform their own oral care, and to studies assessed strategies to prevent or overcome responsive behaviors to oral care. All studies reported improvements of at least some of the outcomes measured, but interpretation is limited due to methodological problems.

**Conclusions:**

Potentially promising strategies are available that nursing home care providers can apply to prevent/overcome residents’ responsive behaviors to oral care or to enable/motivate residents to perform their own oral care. However, studies assessing these strategies have a high risk for bias. To overcome oral health problems in nursing homes, care providers will need practical strategies whose effectiveness was assessed in robust studies.

## Introduction

A significant and growing portion of older adults require long-term care services [1]. Currently, Western countries see 3–8% of the population aged 65 years and older residing in nursing homes [1, 2]. Nursing home residents total almost 225 thousand in Canada [3], 1.3 million in the USA [4], and 2.9 million in Europe [2]. These numbers are expected to increase substantially as the population continues to age [5, 6]. Nursing home residents frequently require partial or complete assistance in conducting activities of daily living, including oral care [2, 4, 7, 8]. However, providing this level of care is often complicated by residents’ cognitive limitations [9]. Between 50% and 75% of nursing home residents have dementia [7, 8, 10–12], and the rate of potentially undetected dementia is over 11% [13]. Currently, there is no effective therapy to prevent, cure or treat dementia, and without dramatic breakthroughs, the global number of people living with dementia (46.8 million in 2015) will almost triple to 131.5 million by 2050 [9]. Complexity of care demands in nursing homes will further increase as persons with dementia stay at home longer with community care and enter nursing homes only at more advanced stages of disease [14, 15]. These demographic shifts highlight a need for proven effective strategies within nursing homes to adequately meet the basic care needs of this vulnerable population.

Poor oral health is frequently seen in nursing home residents as a consequence of inadequate care. Despite professional guidelines for what constitutes proper provision of oral care in older adults [16–19], nursing home residents continue to display less than optimal oral health. Sixty two percent of nursing home residents present with unacceptable levels of oral hygiene [20–22]. Between 44% and 76% of nursing home residents with natural teeth experience dental caries [23–29]. High rates of gingivitis (66%-74%) [26, 29] and periodontitis (32%-49%) [26, 27, 30] are also frequently reported.

Oral conditions have widespread effects on both physical and psychosocial health. Social impacts, such as low self-esteem associated with bad breath or missing/decayed teeth, are prevalent in older adults with poor oral hygiene [31, 32]. Preventable suffering as a result of oral/dental pain can be seen in 3.4%-8% of nursing home residents [26, 27, 30]. Furthermore, poor oral health elevates health care costs and the risk of malnutrition, respiratory infections, diabetes, cardiovascular diseases, and even premature death (e.g., due to aspiration pneumonia) [33–39].

Provision of oral care presents with its own unique challenges. An increasing number of residents are entering facilities with their natural teeth, supported by prostheses such as implants and bridges, which require increased and more complex oral care than previous generations [40]. For example, natural teeth require “in-the-mouth” care, such as brushing and flossing, as opposed to dentures, which simply need to be removed from the oral cavity and then cleaned [16–19]. Dental implants require meticulous care to mitigate the high risks of failure, inflammation, and even bone loss [41]. At the same time, unregulated care aides with little or no formal training provide up to 80% of the direct care (including oral care) in nursing homes [42–44], and both unregulated and regulated care providers receive insufficient training on basic oral care, let alone complex care of various prostheses [45–49]. Regardless of care providers’ oral care knowledge and education, responsive behaviors by residents with dementia are consistently reported as a major barrier to providing adequate oral care [49–52]. Responsive behaviors—defined as physical or verbal actions, such as grabbing, screaming, and resisting care, in response to a negatively perceived stimulus [53, 54]—can make oral care provision time consuming, disruptive, and potentially distressing for the care provider [51]. The term responsive behaviours highlights that those behaviours are meaningful responses to environmental stress or unmet needs rather than just neuropathological symptoms [51, 53, 54]. Additional barriers to providing appropriate oral care in residential facilities include, a low-priority, poorly organized processes and policies, and care providers’ own personal knowledge and attitudes regarding oral health [21, 55, 56].

Researchers have suggested that an enhanced multidisciplinary approach to care, including dentists and dental hygienists, is needed to improve oral health in care facilities [56–58]. While this suggestion has value, interventions and strategies directly targeting front-line care providers are still necessary, as these individuals are responsible for the majority of hands-on daily care, such as tooth brushing [42, 43]. Several reviews have revealed educational interventions as a means to improving oral health [59–61]. These interventions are potentially effective, but study quality is generally low, and heterogeneity of interventions makes best practice recommendations difficult. Furthermore, persons with cognitive impairments, are frequently excluded from these studies, limiting generalizability to a substantial portion of the population in care facilities [51, 59]. Several reviews propose communication strategies to minimize behavioral responses in residents with dementia [62–64]. However, evidence on the effectiveness of these strategies is weak or inconclusive, and these strategies have not been tested in the context of daily oral care. A few specific strategies to reduce responsive behaviors during oral care have been suggested and trialed [65, 66] but to date, no systematic review on the effectiveness of such strategies is available.

In addition to strategies to reduce responsive behaviors, residents and care providers could also benefit from strategies to encourage and motivate residents to complete their own oral care when residents are capable of doing so independently. A quarter of the regularly functioning adult population is not motivated to conduct tooth brushing twice a day [67, 68]. Motivational barriers are further amplified if older adults have low socio-economic status, a history of dental neglect, and generally negative attitudes towards oral care [69–71]. Two systematic reviews have addressed psychological or motivational interventions in order to improve oral care adherence [72, 73]. While included studies were generally of low quality, these reviews provide tentative support that psychological interventions may improve motivation for routine oral care. No reviews have analyzed motivational techniques in the context of long-term care, in which care providers could encourage residents to conduct their own daily oral health care.

In order to provide the best level of oral health care in nursing homes, care providers need to be aware of effective strategies to either: 1) encourage and motivate residents to perform their own oral care, or 2) to prevent and overcome residents’ responsive behaviors so oral care can be adequately provided. The aim of this review is to identify and synthesize evidence on the effectiveness of interventions in nursing homes which provide care providers with such strategies.

## Materials and methods

### Review design

This is a systematic review of quantitative intervention studies. Due to the small number and heterogeneity of included studies we were unable to conduct meta-analyses of study effects. Therefore, we present a narrative synthesis of the available evidence. We registered this study with PROSPERO (CRD42015026439) and published a systematic review protocol [74]. Our methods followed the Cochrane Handbook of Systematic Reviews of Interventions [75] and the Preferred Reporting Items for Systematic Reviews and Meta-Analysis (PRISMA) guidelines [76].

### Search strategy

With a science librarian, we developed, pretested and applied a search strategy (S1 Appendix) combining terms related to oral health with terms related to care providers and residents in nursing homes. On April 8, 2016, we searched the databases Medline, EMBASE, Evidence Based Reviews–Cochrane Central Register of Controlled Trials, CINAHL, and Web of Science. We did not limit language or year of publication, and retrieved all findings starting with the earliest reference available in the respective database. In addition, we searched key journals and key author publications by hand. Based on the number and relevance of published papers, we selected four key journals (*Geriatrics and Gerontology*, *Gerodontology*, *International Journal of Nursing Studies*, *Journal of the American Geriatrics Society*) and ten key authors (Jane M. Chalmers, Ronald L. Ettinger, Marianne Forsell, Rita A. Jablonski, Rie Konno, Michael I. MacEntee, Debora C. Matthews, Mary E. McNally, Inger M. Wårdh, Sheryl Zimmerman). Finally, we screened reference lists of included studies.

### Data management

Using Zotero (https://www.zotero.org/), an open source literature management software that allows online collaboration of researchers, we imported all references identified in the database, then searched and managed these references throughout the review process. We used Zotero to carry out the title and abstract screenings, to attach PDF files of retrieved full texts to the respective references, and to conduct the full text screenings. All review team members received training in using Zotero before the screening process, and we conducted calibration exercises and held regular team meetings to ensure consistency of applying inclusion and exclusion criteria.

### Inclusion and exclusion criteria

Detailed inclusion and exclusion criteria are listed in Table 1. We included ‘gray’ (i.e., not peer reviewed) literature if the publication reported quantitative results assessing effectiveness of an eligible intervention. We included references in any publication language. Language skills of review team members include: English, Chinese (Mandarin and Cantonese), French, German, Korean, and Vietnamese. To assess eligibility of studies published in other languages, we collaborated with our professional contacts and researchers fluent in that language. We included studies conducted in nursing homes (only one of various terms used across countries and jurisdictions to describe these facilities [77]), which we define as facilities that [77–79]:

- mainly accommodate older people with complex health and care needs, who are unable to remain at home or in a supportive living environment
- provide 24-hour support and assistance with activities of daily living and nursing care
- typically deliver health care over an extended time period (often until the resident dies).

**Table 1:** Inclusion and exclusion criteria.

	Inclusion criteria	Exclusion criteria
**Study type**	Primary, empirical, quantitative studies (survey studies, randomized controlled trials, non-randomized trials with or without control group, cohort or case control studies, cross-sectional studies) assessing the effectiveness of an eligible strategy Mixed-methods studies assessing the effectiveness of an eligible strategy quantitatively Systematic reviews and meta-analyses on the effectiveness of an eligible strategy	Non-empirical work (editorials, opinion texts, theoretical discussions) Non-systematic (selective) reviews, qualitative studies (qualitative interviews, focus groups, ethnographic observations, qualitative case studies)
**Reference type**	Articles published in peer reviewed journals ‘Gray’ literature such as articles not peer reviewed, textbooks, reports, and theses as long as they reported quantitative results of a research study	We did not exclude publications based on their reference type
**Publication language**	References published in any language were eligible	We did not exclude references based on publication language
**Intervention**	Strategies that formal care providers can apply to motivate nursing home residents in performing oral health care themselves Strategies that formal care providers can apply to prevent or overcome nursing home residents' responsive behaviours towards oral health care provided by formal care staff	Oral health care tools such as tooth brushes, flossing tape, inter-dental brushes Oral care products such as toothpastes and fluorides products Oral health care techniques such as brushing, flossing, or rinsing
**Control intervention (if applicable)**	Usual care (i.e., no control intervention) Any kind of placebo or comparison intervention (e.g., unspecific communication in the control group versus a specific motivational communication strategy in the intervention group)	Not applicable
**Study outcomes**	Residents’ oral health (e.g., tooth decay, status of dentition, periodontal status, oral hygiene status) Residents’ self-performed oral care (e.g., number of times residents brush or floss teeth, or clean dentures) Residents’ responsive behaviours towards oral care provided by staff (e.g., number of times residents (a) open or refuse to open their mouth, (b) accept or do not accept staff brushing/flossing teeth, (c) accept or do not accept staff taking out or putting back dentures, (d) do or do not express verbal or physical aggression during oral care, or (e) are or are not anxious or nervous during oral care) Staff oral care practices (i.e., proportion of residents on a care unit or in a facility who receive assistance with cleaning their teeth at least once a day, proportion of care aides on a care unit or in a facility who adhere to defined criteria for oral health best practice)	Resident, family member or staff outcomes not related to residents’ oral health or to staff oral care practices
**Setting**	Residential facilities that provide care for frail older adults over a prolonged time period (nursing homes, personal care homes, special or complex care homes, residential long term care facilities, residential facilities, skilled nursing facilities, etc.)	ResidentialResidential facilities providing care for relatively healthy and independent residents (assisted living, supportive living, retirement homes, senior housing) Day or night care facilities Hospitals, home care, primary care, care housing
**Participants**	Formal, paid care providers providing oral care in nursing homes (care aides, registered nurses, licensed practical nurses, dental hygienists, etc.) and Nursing home residents	Unpaid caregivers (family members, friends, volunteers) Students (nursing, dental medicine, dental hygiene, etc.) Managers (care managers, directors of care, facility administrators)

### Study identification

After duplicates were removed, two review team members independently screened titles and abstracts of retrieved studies for inclusion. At all screening steps, reviewers resolved discrepancies in assignment of screened studies by consensus. We retrieved full texts of all included studies and for studies with insufficient information in their titles/abstracts to decide on inclusion. Two review team members screened full texts independently for inclusion. One team member carried out the hand search of key journals and key author publications. A second team member checked the studies included. Two team members independently screened the reference lists of all included studies.

### Quality appraisal

Two review team members independently assessed methodological quality of studies (risk of bias). We discussed results of this step for each study with the full research team and resolved discrepancies by consensus. We applied two validated checklists (S3 Appendix), as appropriate to study design, to assess methodological quality of included studies–each of which were used and described in detail in previous systematic reviews [80–84].

- Clinical studies with or without control group and with or without randomized allocation of participants: Quality Assessment Tool for Quantitative Studies (QATQS) [85]. Reliability and validity of the QATQS have been demonstrated [85, 86]. It assesses the categories of selection bias, study design, confounders, blinding, data collection methods, withdrawals and drop-outs, intervention integrity, and analyses.
- Cross-sectional studies: Estabrooks’ Quality Assessment and Validity Tool for Cross-Sectional Studies. This tool was developed based on Cochrane guidelines [87] and other evidence-based criteria [88, 89]. Reviewers assess methodological quality of studies on 12 items in the categories of sampling, measurement, and statistical analyses.

We rated the overall quality of each study, using a scoring method developed by de Vet et al. [90]. We first calculated the ratio of the obtained score to the maximum possible score, which varies with the checklist used and the number of checklist items applicable. We then used this quality score with a possible range of 0–1, to rank studies as weak (≤0.50), low moderate (0.51–0.66), high moderate (0.67–0.79), or strong (≥0.80).

### Data extraction

One team member extracted the following study details into an Excel spread sheet template: first author, year of publication, title, journal (or type of reference e.g., thesis, report, text book), country of study, study purpose(s), study design, study sample (numbers and types of facilities, care providers, and residents included), strategies studied (including control conditions, if applicable), outcomes assessed (including assessment tools, if applicable), and main results. A second team member double-checked data extraction for each study and discrepancies were resolved by consensus.

### Analyses

We were not able to statistically pool results of included studies, as we could not identify a sufficient number of studies reporting similar designs, methods and outcomes. Therefore, we conducted a narrative synthesis of the included studies. To assess reporting bias, we checked whether a study protocol was published before participants were recruited for each included study, and we compared available study protocols to the published studies.

## Results

### Study selection

**Fig 1:**
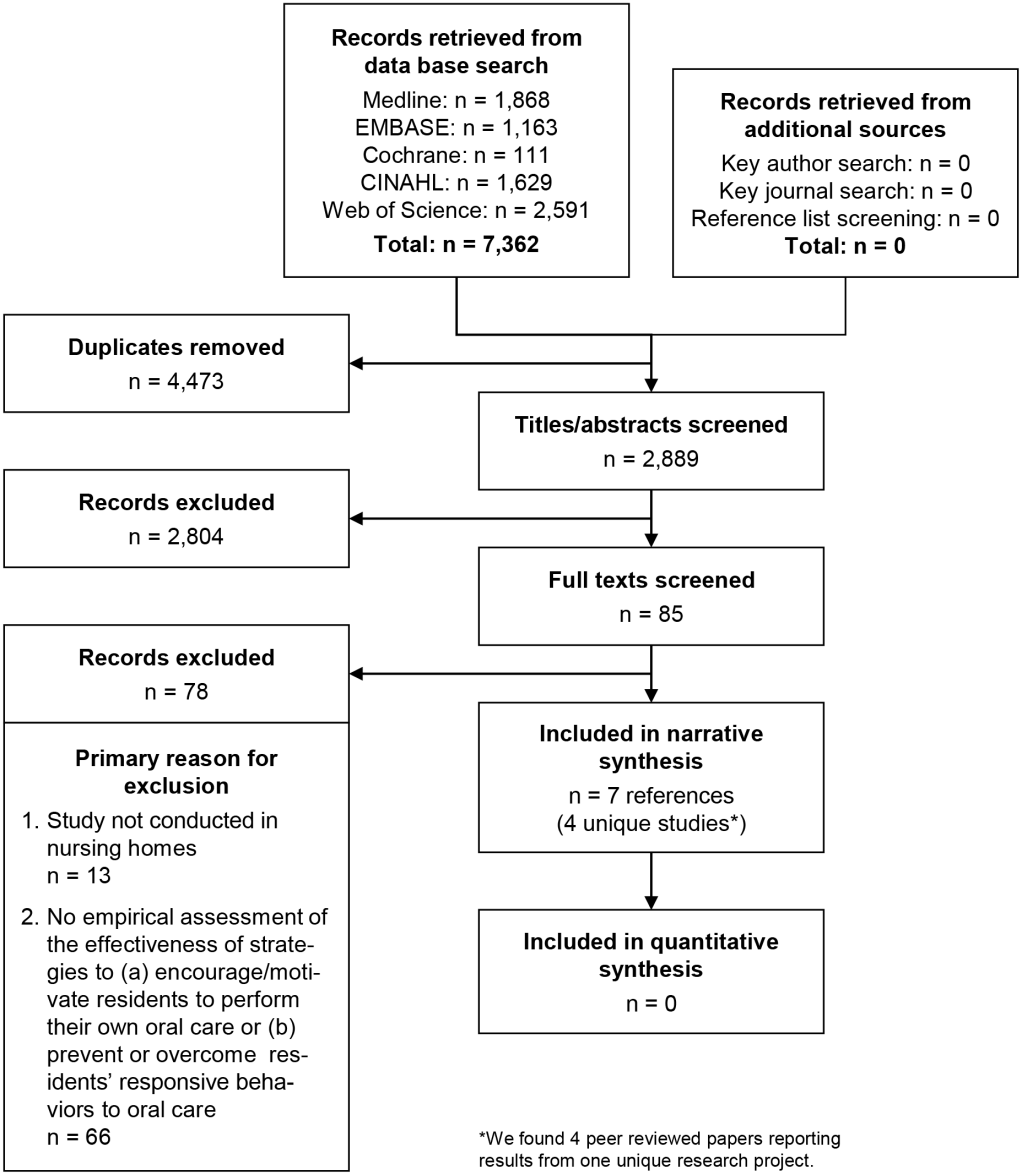
Included and excluded references (modified PRISMA flow diagram).

We included a total of seven references [65, 66, 91–95], four of which report different aspects of one unique research project [66, 92–94]. Therefore, these seven references represent four unique studies (i.e., research projects). Fig 1 (a modified version of the PRISMA flow diagram) details the number of references included and excluded in each step of our review. We did not identify any additional references in our hand search.

### Study characteristics

As Table 2 illustrates, we were not able to identify any randomized trial assessing the effectiveness of any strategy of interest to this review. Three of the included research projects [65, 66, 91–94] were conducted in the USA and applied a one-group pre-test, post-test design, and one was a Canadian cross-sectional study [95]. Methodological quality was low for three of the included research projects [65, 91, 95] and low moderate for one [66, 92–94] (see S4 Appendix for detailed quality ratings).

**Table 2:** Characteristics of included studies.

Study	Country	Design	Study purpose	Sample	Strategies studied	Quality rating
Connell et al. (2002) [91]	USA	One-group pre-post	Illustration of how the physical and social environments of a nursing home can be modified simultaneously, using promotion of greater independence in oral care and adequacy of oral hygiene as a model case. Development of an individualized and revised oral care plan for each resident after observation and assessment to remove barriers to oral health hygiene.	1 nursing home, 5 residents, 1 clinical nurse and various front-line caregivers (no further details reported)	Modifications to the physical environment to compensate for cognitive deficits. Modifications of the physical environment to compensate for other comorbid conditions that could interfere with oral self-care. Instructions to staff regarding how to cue the resident to overcome cognitive deficits and foster use of preserved abilities. Instructions to staff about approaches to care to overcome non-cognitive deficits.	Weak
Jablonski et al. (2011) [65]	USA	One-group pre-post	Testing feasibility of an intervention to reduce care resistant behaviours in persons with moderate-to-severe dementia during oral hygiene activities. Assessment whether reduction in residents’ care resistant behaviours led to improved oral health of residents.	1 nursing home, 7 residents, No details reported on care providers	Approaching resident at eye level and within their visual field Providing care in quiet environment with minimal people Establishing rapport Using gentle touching Smiling during interaction Avoiding elderspeak, or "baby talk" Cueing: using polite one-step commands Gestures and pantomiming Bridging: having resident hold the same item being used in mouth care Distraction Priming: using objects in the environment to initiate or complete mouth care Chaining: having care provider initiate care and expecting the resident to complete task Hand-over-hand: guiding resident's hands Mirror-mirror: providing care in front of the mirror and standing being the resident Rescuing: replacing one care provider with another when behaviors are escalating	Weak
Sloane et al. (2012) [92], Zimmerman et al. (2012) [93], Sloane et al. (2013) [66], Zimmerman et al. (2014) [94]	USA	One-group pre-post	Development and pilot-testing of an evidence-based, person-centered intervention that trains care providers, nurse supervisors, administrators, advocates, and others to better provide oral health care to nursing home residents (especially, but not limited to those with dementia) in order to improve residents' oral health. Preventing or managing responsive behaviours is part of this intervention.	3 nursing homes, 97 residents, 3 certified nursing assistants	Nonspecific Know the person Approach from the front Smile Ask permission before starting Focus on the person rather than the task Explain each step Be patient, repeat yourself as appropriate Give positive feedback and encouragement Establish a routine Person refuses mouth care Figure out why the person is refusing (e.g., bad time, pain, fear) and change approach accordingly Develop a routine (e.g., every day at the same time with the same caregiver) Provide a reason (e.g., let me get the food out of your teeth so you’ll be more comfortable) Phase in mouth care (e.g., do front of teeth one day, back the next, and interdental brush once the person is comfortable) Person won’t open his/her mouth Tell-show-do techniques to promote understanding Touch the mouth, cheek, or jaw with the toothbrush to prompt to open Gently insert toothbrush to cleanse front surfaces of teeth Sing with the person Be patient, try small talk, provide a reason for mouth care Come back at another time when the person might be more responsive Person resists care by grabbing Hand the person the toothbrush and invite to brush Reassure and rub shoulder/arm to help relax Distract or redirect by pausing, singing, talking Hand the person an object to hold and keep hands busy Person bites toothbrush Gently wiggle the toothbrush and ask to open mouth Insert a smaller brush to work around the toothbrush Gently rub cheek to relax jaw muscle Slide finger along the inside of the cheek and massage jaw Person tries to hit or fight caregiver Distract the person (e.g., singing, watching TV) Pick another time of day when the person is calmer (e.g., early morning while sleepy) Stop and come back later Try another caregiver with whom the person is comfortable Check for broken teeth, sore spots, or infection *Person has trouble swallowing*, *or cannot spit* Use a small amount of antimicrobial rinse Use only a pea-sized drop of toothpaste Provide care sitting up Have person tilt head forward and put a cup under the mouth to encourage spitting Avoid swishing Trouble removing or reinserting dentures Ask to open mouth so you can remove/put in their dentures Gently touch the mouth or cheek to prompt to open mouth	Low moderate
Wilson et al. (2013) [95]	Canada	Cross-sectional	Examine formal caregivers' use of communication strategies while assisting residents with moderate and severe Alzheimer's disease during the completion of a basic activity of daily living, specifically toothbrushing.	2 nursing homes, 13 residents, 15 personal support workers	Task-focused communication: verbal strategies such as repetition, open or closed ended question, or negotiation, and non-verbal strategies such as guided touch and pointing Social communication: greetings, compliments miscellaneous strategies: full physical assistance, redirection	Weak

### Types and effectiveness of identified strategies

Two of the included research projects [65, 66, 92–94] assessed strategies to manage responsive behaviors related to oral care (Table 2). The first research project [66, 92–94] assessed these strategies in conjunction with oral health education of care staff. In the second study [65], the trained research team delivered the intervention instead of the care team. The other two studies [91, 95] excluded residents with a history of responsive behaviors to oral care, but focused on strategies to enable and motivate nursing home residents to perform their own oral care. One set of strategies identified involved a modification of the physical environment (e.g., visual cuing/reinforcement by using colored items, mirrors, reminders; placing items within the reach of residents; using ergonomic tooth brushes; or move the over-bed table oral care can be carried out easier). Another set of strategies focused on instructions to staff on how to overcome residents’ cognitive or non-cognitive deficits (e.g., teaching staff how to use a diagram to prompt resident; teaching staff about residents’ preserved abilities and how to elicit them; or teaching staff that a resident may need cues to initiate and stop tasks). A third set of strategies included task focused or social communication, full physical assistance or redirection. As Table 2 shows, there was a large variety of strategies directed at addressing oral care related responsive behaviors.

Table 3 summarizes the effectiveness of identified strategies. Connell et al. [91] reported a reduction of residents’ dental plaque, but due to their small sample size (five residents in one nursing home) the authors performed no statistical significance tests, and interpretation of findings is limited. In a pilot study, Jablonski et al. [65] found a borderline significant (*p* = 0.06) reduction in the average number of residents’ responsive behaviors per minute and significant improvements of oral health scores. Again, only limited conclusions can be drawn due to a small sample size (seven residents in one facility) and other methodological limitations. The intervention tested by Sloane, Zimmerman and colleagues [66, 92–94] improved residents’ dental and denture plaque scores as well as their gingivitis scores. Care providers’ oral care practices improved as well. While a high proportion of care providers already brushed the facial/buccal (outer) teeth surfaces before the intervention (and therefore no significant improvements and could be made), the proportion of residents that had their lingual (inner) surfaces brushed increased significantly after the intervention. Wilson et al. [95] found that encouraging comments and demonstrating an action were significantly correlated with the proportion of completed oral care tasks by residents with moderate dementia. Re-direction was a successful strategy in residents with severe dementia, and full assistance was negatively correlated with task success in this group.

**Table 3:** Effectiveness of identified strategies.

Study	Dependent variable	Independent variable(s)	Method	Findings
Connell et al. (2002) [91]	Plaque index *(nor further details reported)*	Time of assessment *(baseline vs post-intervention)*	Descriptive statistics; no statistical significance tests reported	Improvements in plaque scores ranged from 17% to 83%, depending on the resident; on average (SD) improvement was 47% (27%)
Jablonski et al. (2011) [65]	Resistiveness to Care Scale (modified version) *(number of responsive behaviors per minute)*	Time of assessment *(baseline vs during-intervention)*	Student's t-test for dependent samples	Baseline mean (SD): 2.43 (4.26) Follow-up mean (SD): 1.09 (1.56), *p* = 0.06
	Oral Health Assessment Tool (OHAT) score *(possible range*: *0–16; lower is better)*			Baseline mean (SD): 7.29 (1.25) 7-day follow-up mean (SD): 2.14 (0.90), ***p*<0.001** 14-day follow-up mean (SD): 1.00 (1.26), ***p*<0.001**
Sloane et al. (2012) [92], Zimmerman et al. (2012) [93], Sloane et al. (2013) [66], Zimmerman et al. (2014) [94]	Plaque Index for Long-Term Care *(possible range*: *0–3; lower is better)*	Time of assessment *(baseline vs 8 weeks post intervention)*	Linear mixed models *(random effect*: *resident; fixed effects*: *measurement time*, *facility and measurement time x facility)*	Baseline mean (SD): 2.5 (0.5) Follow-up mean (SD): 1.7 (0.8), ***p*<0.001**
	Gingival Index for Long-Term Care *(possible range*: *0–3; lower is better)*			Baseline mean (SD): 2.9 (0.9) Follow-up mean (SD): 2.1 (0.7), ***p* = 0.04**
	Denture Plaque Index for Long-Term Care *(possible range*: *0–4; lower is better)*			Baseline mean (SD): 1.8 (0.5) Follow-up mean (SD): 1.4 (0.5), ***p*<0.001**
	Percent of intake at meals		General linear mixed models *(random effect*: *resident; fixed effects*: *measurement time*, *facility and measurement time x facility)*	Baseline: 82% Follow-up: 80%, *p* = 0.55
	RAI-MDS 3.0 item: inflamed or bleeding gums *(scored as 0 = not present or 1 = present)*			Baseline: 85.3% Follow-up: 84.5%, *p* = 0.96
	Percent of residents who got outside of sextants brushed			Between 93% and 100% of residents (depending of the sextant) already got the outside of sextants brushed at baseline; Therefore, no significant (*p*>0.05) improvements were seen at follow-up
	Percent of residents who got inside of sextants brushed			Between 33% and 73% of residents (depending on the sextant) got the outside of sextants brushed at baseline; At follow-up, between 88% and 100% of residents (depending on the sextant) got the outside of sextants brushed, ***p*>0.05** for each of the six sextants.
Wilson et al. (2013) [95]	Tooth brushing task success *(percentage of sessions in which residents completed all steps they were given the opportunity to participate in)*	Encouraging comments	Bivariate Pearson correlations	r = 0.837, ***p* = 0.038** (moderate dementia group)
		Demonstrating an action		r = 0.816, ***p* = 0.048** (moderate dementia group)
		Re-direction		r = 0.839, ***p* = 0.018** (severe dementia group)
		Full assistance		r = -0.865, ***p* = 0.012** (severe dementia group)

RAI-MDS = Resident Assessment Instrument–Minimum data Set, SD = standard deviation

## Discussion

Our review is the first of its kind to evaluate the available evidence on the effectiveness of two types of strategies that are highly relevant for care providers when providing oral care to nursing home residents: (a) strategies to prevent or overcome nursing home residents’ responsive behaviors related to oral care, and (b) strategies to encourage and motivate nursing home residents with some self-care capabilities to complete parts of their oral care on their own. Nursing home care providers consistently report residents’ responsive behaviors as one of the most dominant and challenging barriers to providing oral care [49–52]. Supporting residents’ self-care abilities may improve residents’ quality of life [96, 97] and oral health [72, 73]. Multi-component programs to improve oral care in nursing homes often include components like managing residents’ behavioral problems and supporting residents’ self-care abilities [59–61]. However, these components are often not described in sufficient detail and their theoretical and empirical foundation is often unclear [59–61]. Therefore, we were looking for studies that specifically included and described the two types of strategies mentioned above, and assessed their effectiveness.

We found a paucity of evidence related to our research question. Only four research projects assessed the effectiveness of strategies of interest to this review, none of them was a randomized trial, and methodological quality was low or low moderate. One of the included studies was a pilot study [65]. The authors of that study published a study protocol for a randomized trial (the Managing Oral Hygiene Using Threat Reduction Strategies (MOUTh) trial) [98] and a paper describing conceptual foundations of the intervention [51]. While we were able to identify a publication reporting results of the MOUTh trial [99], the publication focused on the delivery of the intervention during the trial (i.e., process evaluation) rather than on the effectiveness of the intervention. At the time of our search (and while writing this manuscript) no publication reporting the effectiveness of the MOUTh intervention was available.

We also identified two related systematic reviews [50, 100] in our search, which included studies that discussed strategies to prevent or manage nursing home residents’ responsive behaviors related to oral care. However, none of the studies included these reviews assessed the effectiveness of these strategies empirically. Therefore, we did not include the two reviews and any of its included studies.

Various studies are available on the effectiveness of strategies to prevent or overcome residents’ responsive behaviors that are not specifically related to oral care situations. In their systematic review Vasse et al. [62] found that communication strategies can be effective when embedded in daily care activities. The review by McGilton et al. [63] confirms these findings. Specifically, the studies included in these reviews suggested that (a) training care aides in *snoezelen* (i.e., multi-sensory stimulation through the use of lighting effects, tactile surfaces, meditative music and the odor of relaxing essential oils [101]) improved residents’ instrumental and affective verbal behavior [102, 103], (b) an educational program for caregivers led to more positive and appropriate interactions demonstrated by residents [104], (c) a staff communication skills program improved residents’ physical and verbal behaviors [105], (d) implementation of individualized care planning improved nurse–resident cooperation [106], and (e) behavior management training for care aides reduced residents’ responsive behaviors [107]. In a systematic review by O’Connor et al., they found that psychosocial interventions can also be potentially effective [64]. They identified the following interventions that had a moderate or large effect on residents’ responsive behaviors: aromatherapy [108, 109], ability-focused education of care staff [104], bed baths [110], and preferred music [111–113]. However, it is unclear from these studies whether such strategies can be effectively applied in the provision of oral care to nursing home residents. For example, essential oils or relaxing music may generally relax residents, but there is no evidence that these relaxed residents are more willing receive oral care from a care provider. Therefore, we need robust studies to assess whether these strategies can be effectively tailored to oral care situations.

Carrying out proper oral hygiene and adhering to oral hygiene instructions is important to prevent oral/dental diseases [72, 73]. Psychological interventions [72] and motivational interviewing [73] have been identified as potentially effective strategies to promote oral care-related behavior change. While these strategies may be effectively applied by nursing home care providers to motivate nursing home residents in improving their oral care practices, these strategies have never been tested in that context, and the available evidence is limited in general. For example, a Cochrane Review on psychological interventions to improve adherence to oral hygiene instructions in adults with periodontal diseases [72] included studies evaluating strategies based on social learning theory [114], cognitive behavioral theory [115], the stages of change model [116], and operant and classical conditioning [117]. The authors found that these interventions were potentially effective in improving plaque scores [114, 115, 117], decreasing gingival bleeding [114], improving self-reported brushing and flossing [114, 115], and increasing self efficacy beliefs concerning flossing [116]. These studies had major methodological limitations and the interventions ignored key aspects of the foundational theories. Furthermore, none of these studies focused on nursing home residents but rather on the general adult population. In a systematic review on the effectiveness of motivational interviewing for improving oral health, Cascaes et al. [73] found conflicting evidence. Motivational interviewing improved tooth brushing in one study [118] but not in another [119]. It also improved fluoride application [120], but not interproximal tooth brushing [118] and dental utilization [121]. While the dental caries improved in one study [120], motivational interviewing had no effect on this outcome in two other studies [119, 121]. Two studies [122, 123] reported improved dental plaque scores and three studies [124–126] did not report improvements in this outcome. Bleeding improved in one study [123] and did not improve in two studies [125, 126]. Motivational interviewing did not improve periodontal probing depth in any of the included studies [118, 123, 125]. Again, the included studies had major methodological limitations and focused on heterogeneous samples other than nursing home residents (e.g., adults in general, or parents of children at different ages). A translation of these strategies to the population of nursing home residents may be possible, but rigorous intervention development and evaluation methods (e.g. following the Medical Research Council guidance [127]) are needed.

### Limitations of this review

The small number of included studies and their limited methodological quality are the major limitations of this review. We were not able to identify any randomized trial. All included studies had a rather small convenience sample (5–97 residents and 1–15 care providers in 1–3 nursing homes), and none of the studies had a control group. Two of the included studies evaluated other strategies (such as staff training in oral health) in conjunction with the strategies of interest to this review. An evaluation of a multi-component program makes it difficult to attribute effects (or lack thereof) to individual components [60]. We did not attempt to contact study authors to obtain unclear study details. Therefore, unreported methodological details may have lowered our quality assessment scores. Due to the low quality and the heterogeneity of methods and outcomes applied by included studies, we were not able to conduct any meta-analyses of the effectiveness of the strategies assessed. Only one research team had a published trial protocol previous to conducting their study [98]. Therefore, we had no way to assess reporting bias for the other studies included. We conducted a comprehensive database and hand search, applying rigorous methods, and included gray literature identified by our search if the reference met our inclusion criteria. However, we did not systematically search all gray literature databases. Therefore, we may have missed relevant work.

## Conclusions

While we were able to identify potentially promising strategies that nursing home care providers can apply to prevent or overcome oral care related responsive behaviors from residents, methodological quality of intervention studies assessing these strategies was low. Other strategies to prevent or overcome care responsive behaviors were never tested in the specific context of oral care provision. We identified an equally big research gap related to strategies that care providers can apply to encourage or motivate nursing home residents in conducting oral care on their own. Psychological strategies directed towards oral care have primarily been tested with study samples other than nursing home residents. Specific tailoring of these strategies to the populations of nursing home residents and care providers, and rigorous effectiveness studies are needed. Without practical strategies that are robustly assessed, care providers will keep struggling with providing proper oral care to nursing home residents, and oral health of nursing home residents will remain a major issue–with severe consequences to residents’ general health and quality of life, as well as, the health care system.

## Supporting information

S1 Appendix Search strategy. (PDF)

S2 Appendix PRISMA checklist. (DOCX)

S3 Appendix Study checklists. (PDF)

S4 Appendix Detailed quality ratings of each included study. (PDF)

## References

[1] Organisation for Economic Co-operation and Development (OECD). OECD health statistics 2015: Long-term care resources and utilisation—long-term care recipients 2015 [February 02, 2017]. http://stats.oecd.org/index.aspx?queryid=30143#.

[2] Commission European. Long-term care for the elderly: provisions and providers in 33 European countries. Luxembourg: Publications Office of the European Union; 2012.

[3] Canada Statistics. Residential Care Facilities 2009/2010. September 2011 ed. Ottawa, Ontario: Statistics Canada; 2011.

[4] Harrington C, Carrillo H, Garfield R. Nursing facilities, staffing, residents and facility deficiencies, 2009 Through 2014. Menlo Park, CA: The Henry J. Kaiser Family Foundation 2015.

[5] Congress of the United States—Congressional Budget Office. Rising demand for long-term services and supports for elderly people. Washington, DC: CBO; 2013.

[6] He W, Goodkind D, Kowal P. An aging world: 2015, international population reports Washington, DC: United States Census Bureau, United States National Institute on Aging; 2016.

[7] Estabrooks CA, Poss JW, Squires JE, Teare GF, Morgan DG, Stewart N, et al A profile of residents in prairie nursing homes. Can J Aging. 2013;32(3):223–31. doi: 10.1017/S0714980813000287 23920244 10.1017/S0714980813000287

[8] Harris-Kojetin L, Sengupta M, Park-Lee E, Valverde R, Caffrey C, Rome V, et al Long-term care providers and services users in the United States: data from the National Study of Long-Term Care Providers, 2013–2014. Vital & health statistics Series 3, Analytical and epidemiological studies. 2016;3(38):x–xii; 1–105. 27023287

[9] Alzheimer's Disease International. World Alzheimer Report 2015: The global impact of dementia—an analysis of prevalence, incidence, cost and trends. London: ADI; 2015.

[10] Hirdes JP, Mitchell L, Maxwell CJ, White N. Beyond the 'iron lungs of gerontology': Using evidence to shape the future of nursing homes in Canada. Can J Aging. 2011;30(3):371–90. doi: 10.1017/S0714980811000304 21851753 10.1017/S0714980811000304

[11] Hoffmann F, Kaduszkiewicz H, Glaeske G, van den Bussche H, Koller D. Prevalence of dementia in nursing home and community-dwelling older adults in Germany. Aging Clin Exp Res. 2014;26(5):555–9. doi: 10.1007/s40520-014-0210-6 24647931 10.1007/s40520-014-0210-6

[12] Stewart R, Hotopf M, Dewey M, Ballard C, Bisla J, Calem M, et al Current prevalence of dementia, depression and behavioural problems in the older adult care home sector: the South East London Care Home Survey. Age and ageing. 2014;43(4):562–7. doi: 10.1093/ageing/afu062 24855111 10.1093/ageing/afu062

[13] Bartfay E, Bartfay WJ, Gorey KM. Prevalence and correlates of potentially undetected dementia among residents of institutional care facilities in Ontario, Canada, 2009–2011. International journal of geriatric psychiatry. 2013;28(10):1086–94. doi: 10.1002/gps.3934 23382109 10.1002/gps.3934

[14] Organisation for Economic Co-operation and Development (OECD). Addressing dementia: The OECD response. Paris: OECD; 2015.

[15] Alzheimer Society of Canada. Rising tide: the impact of dementia on canadian society. Toronto, ON: Alzheimer Society of Canada; 2010.

[16] Registered Nurses' Association of Ontario (RNAO). Oral health: nursing assessment and interventions. Toronto, ON: RNAO, 2008.

[17] De Visschere LM, van der Putten GJ, Vanobbergen JN, Schols JM, de Baat C, Dutch Association of Nursing Home P. An oral health care guideline for institutionalised older people. Gerodontology. 2011;28(4):307–10. 22092286 10.1111/j.1741-2358.2010.00406.x

[18] Johnson VB. Evidence-based practice guideline: oral hygiene care for functionally dependent and cognitively impaired older adults. J Gerontol Nurs. 2012;38(11):11–9. doi: 10.3928/00989134-20121003-02 23126514 10.3928/00989134-20121003-02

[19] O’Connor LJ. Oral health care In: Boltz M, Capezuti E, Fulmer T, Zwicker D, editors. Evidence-based geriatric nursing protocols for best practice. 4. ed ed. New York: Springer; 2012 p. 409–18.

[20] Coleman P, Watson NM. Oral care provided by certified nursing assistants in nursing homes. J Am Geriatr Soc. 2006;54(1):138–43. doi: 10.1111/j.1532-5415.2005.00565.x 16420211 10.1111/j.1532-5415.2005.00565.x

[21] Chami K, Debout C, Gavazzi G, Hajjar J, Bourigault C, Lejeune B, et al Reluctance of Caregivers to Perform Oral Care in Long-Stay Elderly Patients: The Three Interlocking Gears Grounded Theory of the Impediments. J Am Med Dir Assoc. 2012;13(1):e1–e4. doi: 10.1016/j.jamda.2011.06.007 21752721 10.1016/j.jamda.2011.06.007

[22] Zuluaga DJM, Ferreira J, Montoya JAG, Willumsen T. Oral health in institutionalised elderly people in Oslo, Norway and its relationship with dependence and cognitive impairment. Gerodontology. 2012;29(2):e420–e6. doi: 10.1111/j.1741-2358.2011.00490.x 21564272 10.1111/j.1741-2358.2011.00490.x

[23] Wyatt CC. Elderly Canadians residing in long-term care hospitals: Part II. Dental caries status. J Can Dent Assoc. 2002;68(6):359–63. 12034072

[24] Shimazaki Y, Soh I, Koga T, Miyazaki H, Takehara T. Relationship between dental care and oral health in institutionalized elderly people in Japan. J Oral Rehabil. 2004;31(9):837–42. doi: 10.1111/j.1365-2842.2004.01320.x 15369462 10.1111/j.1365-2842.2004.01320.x

[25] Chalmers JM, Carter KD, Fuss JM, Spencer AJ, Hodge CP. Caries experience in existing and new nursing home residents in Adelaide, Australia. Gerodontology. 2002;19(1):30–40. 12164237 10.1111/j.1741-2358.2002.00030.x

[26] Matthews DC, Clovis JB, Brillant MGS, Filiaggi MJ, McNally ME, Kotzer RD, et al Oral health status of long-term care residents: a vulnerable population. J Can Dent Assoc. 2012;78:c3 22364866

[27] Arpin S, Brodeur JM, Corbeil P. Dental caries, problems perceived and use of services among institutionalized elderly in 3 regions of Quebec, Canada. J Can Dent Assoc. 2008;74(9):807-. 19000464

[28] Maupome G, Wyatt CC, Williams PM, Aickin M, Gullion CM. Oral disorders in institution-dwelling elderly adults: a graphic representation. Spec Care Dentist. 2002;22(5):194–200. 12580358 10.1111/j.1754-4505.2002.tb00270.x

[29] Patrick DL, Murray TP, Bigby JA, Auerbach J, Mullen J, Johnson DE, et al The Commonwealth’s high-risk senior population: results and recommendations from 2009 statewide oral health assessment. Boston, MA: Massachusetts Department of Public Health, Office of Oral Health; 2010.

[30] Adegbembo AO, Leake JL, Main PA, Lawrence HL, Chipman ML. The effect of dental insurance on the ranking of dental treatment needs in older residents of Durham Region's homes for the aged. J Can Dent Assoc. 2002;68(7):412–8. 12119091

[31] Locker D, Slade G. Oral health and the quality of life among older adults: the oral health impact profile. J Can Dent Assoc. 1993;59(10):830–3, 7–8, 44 8221283

[32] Slade GD, Spencer AJ, Locker D, Hunt RJ, Strauss RP, Beck JD. Variations in the social impact of oral conditions among older adults in South Australia, Ontario, and North Carolina. J Dent Res. 1996;75(7):1439–50. doi: 10.1177/00220345960750070301 8876595 10.1177/00220345960750070301

[33] Azarpazhooh A, Tenenbaum HC. Separating fact from fiction: Use of high-level evidence from research syntheses to identify diseases and disorders associated with periodontal disease. J Can Dent Assoc. 2012;78:c25 22436432

[34] Haumschild MS, Haumschild RJ. The importance of oral health in long-term care. J Am Med Dir Assoc. 2009;10(9):667–71. doi: 10.1016/j.jamda.2009.01.002 19883892 10.1016/j.jamda.2009.01.002

[35] US Department of Health & Human Services. Healthy People 2010, Volume II (second edition) Washington, DC: US Government Printing Office; 2000 [May 29, 2016]. http://www.healthypeople.gov/2010/Document/tableofcontents.htm#Volume2.

[36] Emami E, de Souza RF, Kabawat M, Feine JS. The impact of edentulism on oral and general health. Int J Dent. 2013;2013:498305 doi: 10.1155/2013/498305 23737789 10.1155/2013/498305 PMC3664508

[37] Awano S, Ansai T, Takata Y, Soh I, Akifusa S, Hamasaki T, et al Oral health and mortality risk from pneumonia in the elderly. J Dent Res. 2008;87(4):334–9. doi: 10.1177/154405910808700418 18362314 10.1177/154405910808700418

[38] Taylor GW, Loesche WJ, Terpenning MS. Impact of oral diseases on systemic health in the elderly: diabetes mellitus and aspiration pneumonia. J Public Health Dent. 2000;60(4):313–20. 11243053 10.1111/j.1752-7325.2000.tb03341.x

[39] Frenkel H, Matthews DC, Nitschke I. Prevention of oral diseases for a dependent population In: MacEntee MI, Müller F, Wyatt CCL, editors. Oral healthcare and the frail elder. Ames, IA: Wiley-Blackwell; 2010 p. 187–209.

[40] McNally ME, Matthews DC, Clovis JB, Brillant M, Filiaggi MJ. The oral health of ageing baby boomers: a comparison of adults aged 45–64 and those 65 years and older. Gerodontology. 2014;31(2):123–35. doi: 10.1111/ger.12022 23216625 10.1111/ger.12022

[41] Louropoulou A, Slot DE, Van der Weijden F. Mechanical self-performed oral hygiene of implant supported restorations: a systematic review. J Evid Based Dent Pract. 2014;14(Suppl. 1):60–9 e1. 24929590 10.1016/j.jebdp.2014.03.008

[42] Berta W, Laporte A, Deber R, Baumann A, Gamble B. The evolving role of health care aides in the long-term care and home and community care sectors in Canada. Hum Resour Health. 2013;11(1):25. 23768158 10.1186/1478-4491-11-25 PMC3723545

[43] Estabrooks CA, Squires JE, Carleton HL, Cummings GG, Norton PG. Who is looking after Mom and Dad? Unregulated workers in Canadian long-term care homes. Can J Aging. 2015;34(1):47–59. doi: 10.1017/S0714980814000506 25525838 10.1017/S0714980814000506 PMC4413363

[44] Bureau of Labor Statistics. Occupational employment statistics: May 2014 national industry-specific occupational employment and wage estimates, NAICS 623100—nursing care facilities (skilled nursing facilities) 2014 [06 Nov 2016]. http://www.bls.gov/oes/current/naics4_623100.htm#29-0000.

[45] Blinkhorn FA, Weingarten L, Boivin L, Plain J, Kay M. An intervention to improve the oral health of residents in an aged care facility led by nurses. Health Educ J. 2012;71(4):527–35.

[46] Young BC, Murray CA, Thomson J. Care home staff knowledge of oral care compared to best practice: a West of Scotland pilot study. Br Dent J. 2008;205(8):E15; discussion 450–1. doi: 10.1038/sj.bdj.2008.894 18841164 10.1038/sj.bdj.2008.894

[47] Preston AJ, Kearns A, Barber MW, Gosney MA. The knowledge of healthcare professionals regarding elderly persons' oral care. Br Dent J. 2006;201(5):293–5; discussion 89; quiz 304. doi: 10.1038/sj.bdj.4813973 16960615 10.1038/sj.bdj.4813973

[48] Vanobbergen JN, De Visschere LM. Factors contributing to the variation in oral hygiene practices and facilities in long-term care institutions for the elderly. Community Dent Health. 2005;22(4):260–5. 16379165

[49] Wardh I, Jonsson M, Wikstrom M. Attitudes to and knowledge about oral health care among nursing home personnel—an area in need of improvement. Gerodontology. 2012;29(2):e787–92. doi: 10.1111/j.1741-2358.2011.00562.x 21950522 10.1111/j.1741-2358.2011.00562.x

[50] Chalmers J, Pearson A. Oral hygiene care for residents with dementia: a literature review. J Adv Nurs. 2005;52(4):410–9. doi: 10.1111/j.1365-2648.2005.03605.x 16268845 10.1111/j.1365-2648.2005.03605.x

[51] Jablonski RA, Therrien B, Kolanowski A. No more fighting and biting during mouth care: applying the theoretical constructs of threat perception to clinical practice. Res Theory Nurs Pract. 2011;25(3):163–75. 22216691 10.1891/1541-6577.25.3.163 PMC3298085

[52] Mancini M, Grappasonni I, Scuri S, Amenta F. Oral health in Alzheimer's disease: a review. Curr Alzheimer Res. 2010;7(4):368–73. 20043813 10.2174/156720510791162359

[53] Speziale J, Black E, Coatsworth-Puspoky R, Ross T, O'Regan T. Moving forward: evaluating a curriculum for managing responsive behaviors in a geriatric psychiatry inpatient population. Gerontologist. 2009;49(4):570–6. doi: 10.1093/geront/gnp069 19520841 10.1093/geront/gnp069

[54] Alzheimer Society Ontario. What are responsive behaviours [2016-02-12]. http://www.alzheimer.ca/en/on/We-can-help/Resources/Shifting-Focus/What-are-responsive-behaviours.

[55] Miegel K, Wachtel T. Improving the oral health of older people in long-term residential care: A review of the literature. Int J Older People Nurs. 2009;4(2):97–113. doi: 10.1111/j.1748-3743.2008.00150.x 20925809 10.1111/j.1748-3743.2008.00150.x

[56] MacEntee MI. Muted dental voices on interprofessional healthcare teams. J Dent. 2011;39 Suppl 2:S34–S40. 22101120 10.1016/j.jdent.2011.10.017

[57] MacEntee MI. Missing links in oral health care for frail elderly people. J Can Dent Assoc. 2006;72(5):421–5. 16772066

[58] Raghoonandan P, Cobban SJ, Compton SM. A scoping review of the use of fluoride varnish in elderly people living in long term care facilities. Can J Dent Hygiene. 2011;45(4):217–22.

[59] Coker E, Ploeg J, Kaasalainen S. The effect of programs to improve oral hygiene outcomes for older residents in long-term care: a systematic review. Res Gerontol Nurs. 2014;7(2):87–100. doi: 10.3928/19404921-20140110-01 24444451 10.3928/19404921-20140110-01

[60] de Lugt-Lustig KH, Vanobbergen JN, van der Putten GJ, De Visschere LM, Schols JM, de Baat C. Effect of oral healthcare education on knowledge, attitude and skills of care home nurses: a systematic literature review. Community Dent Oral Epidemiol. 2014;42(1):88–96. doi: 10.1111/cdoe.12063 23895301 10.1111/cdoe.12063

[61] Weening-Verbree L, Huisman-de Waal G, van Dusseldorp L, van Achterberg T, Schoonhoven L. Oral health care in older people in long term care facilities: A systematic review of implementation strategies. Int J Nurs Stud. 2013;50(4):569–82. doi: 10.1016/j.ijnurstu.2012.12.004 23290098 10.1016/j.ijnurstu.2012.12.004

[62] Vasse E, Vernooij-Dassen M, Spijker A, Rikkert MO, Koopmans R. A systematic review of communication strategies for people with dementia in residential and nursing homes. Int Psychogeriatr. 2010;22(2):189–200. doi: 10.1017/S1041610209990615 19638257 10.1017/S1041610209990615

[63] McGilton KS, Boscart V, Fox M, Sidani S, Rochon E, Sorin-Peters R. A systematic review of the effectiveness of communication interventions for health care providers caring for patients in residential care settings. Worldviews Evid Based Nurs. 2009;6(3):149–59. doi: 10.1111/j.1741-6787.2009.00155.x 19523033 10.1111/j.1741-6787.2009.00155.x

[64] O'Connor DW, Ames D, Gardner B, King M. Psychosocial treatments of behavior symptoms in dementia: a systematic review of reports meeting quality standards. Int Psychogeriatr. 2009;21(2):225–40. doi: 10.1017/S1041610208007588 18814806 10.1017/S1041610208007588

[65] Jablonski RA, Therrien B, Mahoney EK, Kolanowski A, Gabello M, Brock A. An intervention to reduce care-resistant behavior in persons with dementia during oral hygiene: a pilot study. Spec Care Dentist. 2011;31(3):77–87. doi: 10.1111/j.1754-4505.2011.00190.x 21592161 10.1111/j.1754-4505.2011.00190.x

[66] Sloane PD, Zimmerman S, Chen X, Barrick AL, Poole P, Reed D, et al Effect of a person-centered mouth care intervention on care processes and outcomes in three nursing homes. J Am Geriatr Soc. 2013;61(7):1158–63. doi: 10.1111/jgs.12317 23772769 10.1111/jgs.12317

[67] Health Canada. Report on the findings of the oral health component of the Canadian Health Measures Survey 2007–2009. Ottawa: Health Canada; 2010.

[68] Ganss C, Schlueter N, Preiss S, Klimek J. Tooth brushing habits in uninstructed adults—frequency, technique, duration and force. Clin Oral Investig. 2009;13(2):203–8. doi: 10.1007/s00784-008-0230-8 18853203 10.1007/s00784-008-0230-8

[69] Institute of Medicine of the National Academies. Advancing oral health in America. Washington, DC: The National Academies Press; 2011.

[70] Yao CS, MacEntee MI. Inequity in oral health care for elderly Canadians: part 3. Reducing barriers to oral care. J Can Dent Assoc. 2014;80:e11. 24598327

[71] McGrath C, Zhang W, Lo EC. A review of the effectiveness of oral health promotion activities among elderly people. Gerodontology. 2009;26(2):85–96. doi: 10.1111/j.1741-2358.2008.00232.x 19490131 10.1111/j.1741-2358.2008.00232.x

[72] Renz A, Ide M, Newton T, Robinson PG, Smith D. Psychological interventions to improve adherence to oral hygiene instructions in adults with periodontal diseases. Cochrane Database Syst Rev. 2007;2007(2):Cd005097. 10.1002/14651858.CD005097.pub2 17443571

[73] Cascaes AM, Bielemann RM, Clark VL, Barros AJ. Effectiveness of motivational interviewing at improving oral health: a systematic review. Rev Saude Publica. 2014;48(1):142–53. doi: 10.1590/S0034-8910.2014048004616 24789647 10.1590/S0034-8910.2014048004616 PMC4206116

[74] Hoben M, Kent A, Kobagi N, Yoon MN. Effective strategies to motivate nursing home residents in oral healthcare and to prevent or reduce responsive behaviours to oral healthcare: a systematic review protocol. BMJ Open. 2016;6(3):e011159 doi: 10.1136/bmjopen-2016-011159 27013601 10.1136/bmjopen-2016-011159 PMC4809102

[75] Higgins JPT, Green S, editors. Cochrane handbook for systematic reviews of interventions Version 5.1.0 [updated March 2011]: The Cochrane Collaboration; 2015.

[76] Moher D, Liberati A, Tetzlaff J, Altman DG, Group P. Preferred reporting items for systematic reviews and meta-analyses: the PRISMA statement. PLoS Med. 2009;6(7):e1000097 doi: 10.1371/journal.pmed.1000097 19621072 10.1371/journal.pmed.1000097 PMC2707599

[77] McGregor MJ, Ronald LA. Residential long-term care for canadian seniors: nonprofit, for-profit or does it matter? Montreal, QC: Institute for Research on Public Policy; 2011.

[78] Jansen I, Murphy J. Residential long-term care in Canada: our vision for better seniors’ care. Ottawa, ON: Canadian Union of Public Employees; 2009.

[79] Canadian Healthcare Association. New directions for facility-based long term care. Ottawa, ON: Canadian Healthcare Association; 2009.

[80] Kajermo KN, Boström AM, Thompson DS, Hutchinson AM, Estabrooks CA, Wallin L. The BARRIERS scale—The barriers to research utilization scale: A systematic review. Implement Sci. 2010;5(1):32. 20420696 10.1186/1748-5908-5-32 PMC2883534

[81] Squires J, Estabrooks C, Gustavsson P, Wallin L. Individual determinants of research utilization by nurses: a systematic review update. Implement Sci. 2011;6(1):1. 21208425 10.1186/1748-5908-6-1 PMC3024963

[82] Squires JE, Hutchinson AM, Boström AM, O'Rourke HM, Cobban SJ, Estabrooks CA. To what extent do nurses use research in clinical practice? A systematic review. Implement Sci. 2011;6(1):21. 21414206 10.1186/1748-5908-6-21 PMC3068972

[83] Squires JE, Hoben M, Linklater S, Carleton HL, Estabooks CA. Job satisfaction among care aides in residential long-term care: A systematic review of contributing factors, both individual and organizational. Nurs Res Pract. 2015;2015(Article ID 157924). 10.1155/2015/157924 PMC4541006 26345545

[84] Hoben M, Buscher I, Berendonk C, Quasdorf T, Riesner C, Wilborn D. Scoping review of nursing-related dissemination and implementation research in German-speaking countries: mapping the field. Int J Health Prof. 2014;1(1):34–49.

[85] Thomas BH, Ciliska D, Dobbins M, Micucci S. A process for systematically reviewing the literature: providing the research evidence for public health nursing interventions. Worldviews Evid Based Nurs. 2004;1(3):176–84. doi: 10.1111/j.1524-475X.2004.04006.x 17163895 10.1111/j.1524-475X.2004.04006.x

[86] Armijo-Olivo S, Stiles CR, Hagen NA, Biondo PD, Cummings GG. Assessment of study quality for systematic reviews: a comparison of the Cochrane Collaboration Risk of Bias Tool and the Effective Public Health Practice Project Quality Assessment Tool: methodological research. J Eval Clin Pract. 2012;18(1):12–8. doi: 10.1111/j.1365-2753.2010.01516.x 20698919 10.1111/j.1365-2753.2010.01516.x

[87] Clarke M, Oxman AD, editors. Cochrane Reviewers' Handbook 4.1.4 (October 2001). Oxford, UK: The Cochrane Library; 2001.

[88] Kmet L, Lee R, Cook L. Standard quality assessment criteria for evaluating primary research papers from a variety of fields. Edmonton, AB: Heritage Foundation for Medical Research; 2004.

[89] Khan KS, ter Riet G, Popay J, Nixon J, Kleijnen J. Stage II conducting the review: Phase 5 study quality assessment. In: Centre of Reviews and Dissemination UoY, editor. Undertaking systematic reviews of research effectiveness CDC’s guidance for those carrying out or commissioning reviews2001. p. 1–20.

[90] de Vet HCW, de Bie RA, van der Heijden GJMG, Verhagen AP, Sijpkes P, Knipschild PG. Systematic reviews on the basis of methodological criteria. Physiotherapy. 1997;83(6):284–9.

[91] Connell BR, McConnell ES, Francis TG. Tailoring the environment of oral health care to the needs and abilities of nursing home residents with dementia. Alzheimer's Care Quarterly. 2002;3(1):19–25.

[92] Sloane P, Chen X, Cohen L, Barrick AL, Poole P, Zimmerman S. Oral health outcomes of person-centered mouth care for persons with cognitive or physical impairment: Mouth care without a battle. Alzheimer's & Dementia: The Journal of the Alzheimer's Association. 2012;8(4):P251–P2.

[93] Zimmerman S, Cohen L, Barrick AL, Sloane P. Implementation of personalized, evidence-based mouth care for persons with cognitive or physical impairment: Mouth care without a battle. Alzheimer's & Dementia: The Journal of the Alzheimer's Association. 2012;8(4):P384.

[94] Zimmerman S, Sloane PD, Cohen LW, Barrick AL. Changing the culture of mouth care: mouth care without a battle. Gerontologist. 2014;54(Suppl1):S25–34. 24443603 10.1093/geront/gnt145

[95] Wilson R, Rochon E, Mihailidis A, Leonard C. Quantitative analysis of formal caregivers' use of communication strategies while assisting individuals with moderate and severe Alzheimer's disease during oral care. J Commun Disord. 2013;46(3):249–63. doi: 10.1016/j.jcomdis.2013.01.004 23523100 10.1016/j.jcomdis.2013.01.004

[96] Shippee TP, Henning-Smith C, Kane RL, Lewis T. Resident- and Facility-Level Predictors of Quality of Life in Long-Term Care. Gerontologist. 2015;55(4):643–55. doi: 10.1093/geront/gnt148 24352532 10.1093/geront/gnt148 PMC4542585

[97] Beerens HC, Zwakhalen SM, Verbeek H, Ruwaard D, Ambergen AW, Leino-Kilpi H, et al Change in quality of life of people with dementia recently admitted to long-term care facilities. J Adv Nurs. 2015;71(6):1435–47. doi: 10.1111/jan.12570 25403506 10.1111/jan.12570

[98] Jablonski RA, Kolanowski A, Therrien B, Mahoney EK, Kassab C, Leslie DL. Reducing care-resistant behaviors during oral hygiene in persons with dementia. BMC Oral Health. 2011;11(1):30. 22100010 10.1186/1472-6831-11-30 PMC3231974

[99] Jablonski-Jaudon RA, Kolanowski AM, Winstead V, Jones-Townsend C, Azuero A. Maturation of the MOUTh Intervention: From Reducing Threat to Relationship-Centered Care. J Gerontol Nurs. 2016;42(3):15–23; quiz 4–5. doi: 10.3928/00989134-20160212-05 26934969 10.3928/00989134-20160212-05 PMC4861900

[100] Pearson A, Chalmers J. Oral hygiene care for adults with dementia in residential aged care facilities. JBI Reports. 2004;2(3):65–113. 10.11124/01938924-200402030-00001 27820001

[101] Chung JC, Lai CK, Chung PM, French HP. Snoezelen for dementia. Cochrane Database Syst Rev. 2002(4):Cd003152 doi: 10.1002/14651858.CD003152 12519587 10.1002/14651858.CD003152 PMC9002239

[102] van Weert JC, van Dulmen AM, Spreeuwenberg PM, Ribbe MW, Bensing JM. Effects of snoezelen, integrated in 24 h dementia care, on nurse-patient communication during morning care. Patient Educ Couns. 2005;58(3):312–26. doi: 10.1016/j.pec.2004.07.013 16054329 10.1016/j.pec.2004.07.013

[103] van Weert JC, Janssen BM, van Dulmen AM, Spreeuwenberg PM, Bensing JM, Ribbe MW. Nursing assistants' behaviour during morning care: effects of the implementation of snoezelen, integrated in 24-hour dementia care. J Adv Nurs. 2006;53(6):656–68. doi: 10.1111/j.1365-2648.2006.03772.x 16553674 10.1111/j.1365-2648.2006.03772.x

[104] Wells DL, Dawson P, Sidani S, Craig D, Pringle D. Effects of an abilities-focused program of morning care on residents who have dementia and on caregivers. J Am Geriatr Soc. 2000;48(4):442–9. 10798473 10.1111/j.1532-5415.2000.tb04704.x

[105] McCallion P, Toseland RW, Lacey D, Banks S. Educating nursing assistants to communicate more effectively with nursing home residents with dementia. Gerontologist. 1999;39(5):546–58. 10568079 10.1093/geront/39.5.546

[106] Edberg AK, Hallberg IR. Effects of clinical supervision on nurse-patient cooperation quality: a controlled study in dementia care. Clin Nurs Res. 1996;5(2):127–46; discussion 47–9. doi: 10.1177/105477389600500202 8704662 10.1177/105477389600500202

[107] Burgio LD, Stevens A, Burgio KL, Roth DL, Paul P, Gerstle J. Teaching and maintaining behavior management skills in the nursing home. Gerontologist. 2002;42(4):487–96. 12145376 10.1093/geront/42.4.487

[108] Ballard CG, O'Brien JT, Reichelt K, Perry EK. Aromatherapy as a safe and effective treatment for the management of agitation in severe dementia: the results of a double-blind, placebo-controlled trial with Melissa. J Clin Psychiatry. 2002;63(7):553–8. 12143909 10.4088/jcp.v63n0703

[109] Holmes C, Hopkins V, Hensford C, MacLaughlin V, Wilkinson D, Rosenvinge H. Lavender oil as a treatment for agitated behaviour in severe dementia: a placebo controlled study. Int J Geriatr Psychiatry. 2002;17(4):305–8. doi: 10.1002/gps.593 11994882 10.1002/gps.593

[110] Dunn JC, Thiru-Chelvam B, Beck CH. Bathing. Pleasure or pain? J Gerontol Nurs. 2002;28(11):6–13. 12465197 10.3928/0098-9134-20021101-05

[111] Clark ME, Lipe AW, Bilbrey M. Use of music to decrease aggressive behaviors in people with dementia. J Gerontol Nurs. 1998;24(7):10–7. 9801526 10.3928/0098-9134-19980701-05

[112] Gerdner LA. Effects of individualized versus classical "relaxation" music on the frequency of agitation in elderly persons with Alzheimer's disease and related disorders. Int Psychogeriatr. 2000;12(1):49–65. 10798453 10.1017/s1041610200006190

[113] Garland K, Beer E, Eppingstall B, O'Connor DW. A comparison of two treatments of agitated behavior in nursing home residents with dementia: simulated family presence and preferred music. Am J Geriatr Psychiatry. 2007;15(6):514–21. doi: 10.1097/01.JGP.0000249388.37080.b4 17293386 10.1097/01.JGP.0000249388.37080.b4

[114] Little SJ, Hollis JF, Stevens VJ, Mount K, Mullooly JP, Johnson BD. Effective group behavioral intervention for older periodontal patients. J Periodontal Res. 1997;32(3):315–25. 9138198 10.1111/j.1600-0765.1997.tb00540.x

[115] Stewart JE, Jacobs-Schoen M, Padilla MR, Maeder LA, Wolfe GR, Hartz GW. The effect of a cognitive behavioral intervention on oral hygiene. J Clin Periodontol. 1991;18(4):219–22. 1856301 10.1111/j.1600-051x.1991.tb00418.x

[116] Stewart JE, Wolfe GR, Maeder L, Hartz GW. Changes in dental knowledge and self-efficacy scores following interventions to change oral hygiene behavior. Patient Educ Couns. 1996;27(3):269–77. 8788355 10.1016/0738-3991(95)00843-8

[117] Weinstein R, Tosolin F, Ghilardi L, Zanardelli E. Psychological intervention in patients with poor compliance. J Clin Periodontol. 1996;23(3 Pt 2):283–8. 8707991 10.1111/j.1600-051x.1996.tb02090.x

[118] Lopez-Jornet P, Fabio CA, Consuelo RA, Paz AM. Effectiveness of a motivational-behavioural skills protocol for oral hygiene among patients with hyposalivation. Gerodontology. 2014;31(4):288–95. doi: 10.1111/ger.12037 23480201 10.1111/ger.12037

[119] Ismail AI, Ondersma S, Jedele JM, Little RJ, Lepkowski JM. Evaluation of a brief tailored motivational intervention to prevent early childhood caries. Community Dent Oral Epidemiol. 2011;39(5):433–48. doi: 10.1111/j.1600-0528.2011.00613.x 21916925 10.1111/j.1600-0528.2011.00613.x PMC3177165

[120] Harrison R, Benton T, Everson-Stewart S, Weinstein P. Effect of motivational interviewing on rates of early childhood caries: a randomized trial. Pediatr Dent. 2007;29(1):16–22. 18041508

[121] Harrison RL, Veronneau J, Leroux B. Effectiveness of maternal counseling in reducing caries in Cree children. J Dent Res. 2012;91(11):1032–7. doi: 10.1177/0022034512459758 22983408 10.1177/0022034512459758

[122] Godard A, Dufour T, Jeanne S. Application of self-regulation theory and motivational interview for improving oral hygiene: a randomized controlled trial. J Clin Periodontol. 2011;38(12):1099–105. doi: 10.1111/j.1600-051X.2011.01782.x 22092542 10.1111/j.1600-051X.2011.01782.x

[123] Jonsson B, Ohrn K, Lindberg P, Oscarson N. Evaluation of an individually tailored oral health educational programme on periodontal health. J Clin Periodontol. 2010;37(10):912–9. doi: 10.1111/j.1600-051X.2010.01590.x 20561115 10.1111/j.1600-051X.2010.01590.x

[124] Almomani F, Williams K, Catley D, Brown C. Effects of an oral health promotion program in people with mental illness. J Dent Res. 2009;88(7):648–52. doi: 10.1177/0022034509338156 19605879 10.1177/0022034509338156

[125] Brand VS, Bray KK, MacNeill S, Catley D, Williams K. Impact of single-session motivational interviewing on clinical outcomes following periodontal maintenance therapy. Int J Dent Hyg. 2013;11(2):134–41. doi: 10.1111/idh.12012 23279918 10.1111/idh.12012

[126] Stenman J, Lundgren J, Wennstrom JL, Ericsson JS, Abrahamsson KH. A single session of motivational interviewing as an additive means to improve adherence in periodontal infection control: a randomized controlled trial. J Clin Periodontol. 2012;39(10):947–54. doi: 10.1111/j.1600-051X.2012.01926.x 22845421 10.1111/j.1600-051X.2012.01926.x

[127] Craig P, Dieppe P, Macintyre S, Michie S, Nazareth I, Petticrew M. Developing and evaluating complex interventions: the new Medical Research Council guidance. Int J Nurs Stud. 2013;50(5):587–92. doi: 10.1016/j.ijnurstu.2012.09.010 23159157 10.1016/j.ijnurstu.2012.09.010

