# Supplementary material for: Effective strategies to motivate nursing home residents in oral care and to prevent or reduce responsive behaviors to oral care: A systematic review
Source: PLoS One. 2017 Jun 13;12(6):e0178913. doi: 10.1371/journal.pone.0178913 (PMC5469468; doi:10.1371/journal.pone.0178913)
Supplement: S1 Appendix — (PDF) [file pone.0178913.s001.pdf]

**S1 Appendix: Search strategy**

**MEDLINE 1946 to Present, MEDLINE(R) In-Process & Other Non-Indexed Citations, EBM Reviews - Cochrane Central Register of Controlled Trials (platform OVID)**

| | |
|---|---|
| 1. | exp Dentistry/ |
| 2. | exp Tooth Diseases/ |
| 3. | Oral Health/ |
| 4. | Oral Hygiene/ |
| 5. | ((oral or dental or mouth) adj (health or care or hygiene)).mp. |
| 6. | or/1-5 |
| 7. | Geriatric Nursing/ or nursing homes/ or Intermediate Care Facilities/ or skilled nursing facilities/ or homes for the aged/ or "Institutionalization"/ |
| 8. | (nursing adj (home* or center* or centre* or facilit*)).tw. |
| 9. | ((extended or long term or intermediate or skilled) adj care).tw. |
| 10. | ((extended or long term or intermediate or skilled) adj facilit*).tw. |
| 11. | ((elderly or senior* or geriatric or veteran*) adj3 (institution* or home* or facilit* or unit* or center* or centre*)).tw. |
| 12. | (rest adj2 home*).tw. |
| 13. | convalescen* home*.tw. |
| 14. | assisted care facilit*.tw. |
| 15. | continuing care.tw. |
| 16. | residential care.tw. |
| 17. | or/7-16 |
| 18. | 6 and 17 |
| 19. | exp Child/ |
| 20. | (child* or boy* or girl* or p?ediatric* or teen* or youth* or adolescen*).mp. |
| 21. | 19 or 20 |
| 22. | exp aged/ |
| 23. | (senior* or elder* or geriatric* or gerontolog*).mp. |
| 24. | 22 or 23 |
| 25. | 21 and 24 |
| 26. | 21 not 25 |
| 27. | 18 not 26 |
| 28. | 27 or 6 |
| 29. | remove duplicates from 28 |

**CINAHL (platform EBSCOhost)**

| | |
|---|---|
| S1 | (MH "Dentistry+") OR (MH "Tooth Diseases+") OR (MH "Oral Health") OR (MH "Oral Hygiene+") OR (MH "Dental Hygiene") |
| S2 | ( oral W0 (health or care or hygiene) ) OR ( dental W0 (health or care or hygiene) ) OR ( mouth W0 (health or care or hygiene) ) |
| S3 | S1 OR S2 |
| S4 | (MH "Gerontologic Nursing+") OR (MH "Nursing Homes+") |
| S5 | (MH "Nursing Home Patients") OR (MH "Institutionalization+") |
| S6 | nursing W0 (home* or center* or centre* or facilit*) |
| S7 | "extended care" or "long term care" or "intermediate care" or "skilled care" |
| S8 | (extended or "long term" or intermediate or skilled) W2 facilit* |
| S9 | ( (elderly or senior* or geriatric or veteran*) N3 institution* ) OR ( (elderly or senior* or geriatric or veteran*) N3 home* ) OR ( (elderly or senior* or geriatric or veteran*) N3 facilit* ) OR ( (elderly or senior* or geriatric or veteran*) N3 unit* ) OR ( (elderly or senior* or geriatric or veteran*) N3 center* ) OR ( (elderly or senior* or geriatric or veteran*) N3 centre* ) |
| S10 | "rest home*" OR "convalescen* home*" OR "assisted care facilit*" OR :continuing care" OR "residential care" |
| S11 | S4 OR S5 OR S6 OR S7 OR S8 OR S9 OR S10 |
| S12 | S3 AND S11 |
| S13 | S3 AND S11<br>Limiters - Research Article |
| S14 | senior* or older* or gerontolog* or geriatric* or elder* |
| S15 | S13 AND S14 |
| S16 | S3 AND S11 |
| S17 | S15 OR S16 |

**Web of Science Core Collection**

| | |
|---|---|
| #1 | TS=("oral health" OR "oral care" OR "oral hygiene" OR dentistry OR "dental health" OR "dental care" OR "dental hygiene") |
| #2 | TS=("nursing home*" or "residential care" or "continuing care" or facilit* or residence or residential or "long term care" or institution*) |
| #3 | #1 AND #2 |
