# Supplementary material for: Effective strategies to motivate nursing home residents in oral care and to prevent or reduce responsive behaviors to oral care: A systematic review
Source: PLoS One. 2017 Jun 13;12(6):e0178913. doi: 10.1371/journal.pone.0178913 (PMC5469468; doi:10.1371/journal.pone.0178913)
Supplement: S3 Appendix — (PDF) [file pone.0178913.s003.pdf]

S2 Appendix: Checklists used to assess methodological quality (risk for bias) of included studies)

1. **Quality Assessment Tool for Quantitative Studies (QATQS)**
   *Required

Reviewer Information

1. Name of Reviewer *

....

2. **First or second review** *

Tick all that apply.

- [ ] First review
- [ ] Second review

General Study Information

3. Study Title *

....

4. Name of First Author *

....

5. Year of Publication *

6. Journal *

For references not published in a journal enter if it is a textbook, report, thesis, etc.

....

A) Selection Bias

7. (Q1) Are the individuals selected to participate in the study likely to be representative of the target population? *

Tick all that apply.

- [ ] Very likely
- [ ] Somewhat likely
- [ ] Not likely
- [ ] Can't tell

8. (Q2) What percentage of selected individuals agreed to participate? *

Tick all that apply.

- [ ] 80 - 100% agreement
- [ ] 60 - 79% agreement
- [ ] Less than 60% agreement
- [ ] Not applicable
- [ ] Can't tell

9. Overall rating of this section *

See dictionary

Tick all that apply.

- [ ] Strong
- [ ] Moderate
- [ ] Weak

B) Study Design

10. Indicate the study design *

Tick all that apply.

- [ ] Randomized controlled trial
- [ ] Controlled clinical trial
- [ ] Cohort analytic (two group pre + post)
- [ ] Case-control
- [ ] Cohort (one group pre + post (before after))
- [ ] Interrupted time series
- [ ] Can't tell
- [ ] Other: ______________________

11. Was study described as randomized? *

If 'No', go to Component C

Tick all that apply.

- [ ] No
- [ ] Yes

12. If 'Yes', was the method of randomization described? *

See dictionary

Tick all that apply.

- [ ] No
- [ ] Yes
- [ ] Not applicable (if 'No' to question 'Was study described as randomized?')

13. If 'Yes', was the method appropriate? *

See dictionary

Tick all that apply.

- [ ] No
- [ ] Yes
- [ ] Not applicable (if 'No' to question 'Was study described as randomized?')

14. Overall rating of this section *

See dictionary

Tick all that apply.

- [ ] Strong
- [ ] Moderate
- [ ] Weak

C) Confounders

***15.* (Q1) Were there important differences between groups prior to the intervention? ***

Tick all that apply.

- [ ] Yes
- [ ] No
- [ ] Can't tell

16. The following are examples of confounders: *

Check all that apply

Tick all that apply.

- [ ] Race
- [ ] Sex
- [ ] Marital status/family
- [ ] Age
- [ ] SES (income or class)
- [ ] Education
- [ ] Health status
- [ ] Pre-intervention score on outcome measure
- [ ] Other: ______________________

17. **(Q2) If yes, indicate the percentage of relevant confounders that were controlled (either in the design (e.g. stratification, matching) or analysis)? ***

Tick all that apply.

- [ ] 80 - 100% (most)
- [ ] 60 - 79% (some)
- [ ] Less than 60% (few or none)
- [ ] Can't tell

18. **Overall rating of this section ***

See dictionary
Tick all that apply.

- [ ] Strong
- [ ] Moderate
- [ ] Weak

D) Blinding

19. **(Q1) Was (were) the outcome assessor(s) aware of the intervention or exposure status of participants? ***

Tick all that apply.

- [ ] Yes
- [ ] No
- [ ] Can't tell

20. **(Q2) Were the study participants aware of the research question? ***

Tick all that apply.

- [ ] Yes
- [ ] No
- [ ] Can't tell

21. **Overall rating of this section ***

See dictionary
Tick all that apply.

- [ ] Strong
- [ ] Moderate
- [ ] Weak

E) Data Collection Methods

22. (Q1) Were data collection tools shown to be valid? *

Tick all that apply.

- [ ] Yes
- [ ] No
- [ ] Can't tell

23. (Q2) Were data collection tools shown to be reliable? *

Tick all that apply.

- [ ] Yes
- [ ] No
- [ ] Can't tell

24. Overall rating of this section *

See dictionary

Tick all that apply.

- [ ] Strong
- [ ] Moderate
- [ ] Weak

F) Withdrawals and Drop-Outs

25. (Q1) Were withdrawals and drop-outs reported in terms of numbers and/or reasons per group? *

Tick all that apply.

- [ ] Yes
- [ ] No
- [ ] Can't tell
- [ ] Not applicable (i.e., one time surveys or interviews)

26. (Q2) Indicate the percentage of participants completing the study. (If the percentage differs by groups, record the lowest). *

Tick all that apply.

- [ ] 80 - 100%
- [ ] 60 - 79%
- [ ] Less than 60%
- [ ] Can't tell
- [ ] Not applicable (i.e., retrospective case-control)

27. **Overall rating of this section** *

See dictionary

Tick all that apply.

- [ ] Strong
- [ ] Moderate
- [ ] Weak
- [ ] Not Applicable

G) Intervention Integrity

28. **(Q1) What percentage of participants received the allocated intervention or exposure of interest?** *

Tick all that apply.

- [ ] 80 - 100%
- [ ] 60 - 79%
- [ ] Less than 60%
- [ ] Can't tell

29. **(Q2) Was the consistency of the intervention measured?**

Tick all that apply.

- [ ] Yes
- [ ] No
- [ ] Can't tell

30. **(Q3) Is it likely that subjects received an unintended intervention (contamination or co-intervention) that may influence the results?** *

Tick all that apply.

- [ ] Yes
- [ ] No
- [ ] Can't tell

H) Analyses

31. **(Q1) Indicate the unit of allocation** *

Tick all that apply.

- [ ] Community
- [ ] Organization/institution
- [ ] Practice/office
- [ ] Individual

32. (Q2) Indicate the unit of analysis *
Tick all that apply.

- [ ] Community
- [ ] Organization/institution
- [ ] Practice/office
- [ ] Individual

33. (Q3) Are the statistical methods appropriate for the study design? *
Tick all that apply.

- [ ] Yes
- [ ] No
- [ ] Can't tell

34. (Q4) Is the analysis performed by intervention allocation status (i.e. intention to treat) rather than the actual intervention received? *
Tick all that apply.

- [ ] Yes
- [ ] No
- [ ] Can't tell

Global Rating For This Paper

For this global rating refer to the overall ratings of sections A-F

35. *
Tick all that apply.

- [ ] Strong (no weak ratings)
- [ ] Moderate (one weak rating)
- [ ] Weak (two or more weak ratings)

2. Estabrooks' Quality Assessment and Validity Tool for Cross-Sectional Studies

*Required

Reviewer Information

1. Name of Reviewer *

....

2. **First or second review?**

* *Check all that apply.*

- [ ] First review
- [ ] Second review

General Study Information

3. Study Title *

....

4. Name of First Author *

....

5. Year of Publication *

6. Journal *

For references not published in a journal enter if it is a textbook, report, thesis, etc.

....

Sampling

N/A can only be selected if the respective item is not applicable to the design of the study

1. **Was probability sampling used? ***

   Most researchers probably used a convenience sample, i.e., studying all the nurses available to them in one or more setting(s) that agreed to participate (which would be the option 'No'). Select 'Yes' if the authors stated that they used a probabilistic sample. Select 'No' if the authors stated that they used a convenience sample or if they did not report the use of probabilistic sample.

   Check all that apply.

   - [ ] Yes
   - [ ] No

2. **Are the individuals selected to participate in the study likely to be representative of the target population?***

   Select 'Very Likely' if the authors have done everything reasonably possible to ensure that the target population is represented. Select 'Somewhat Likely' if participants may not be representative (i.e., if they are referred from a source within a target population even if it is in a systematic manner. Select 'Not Likely' if participants are probably not representative if they are self-referred or are volunteers or if you can not tell.

   Check all that apply.

   - [ ] Very likely
   - [ ] Somewhat likely
   - [ ] Not likely

3. **Was sample size justified to obtain appropriate power? ***

   Select 'Yes' if one or more of the following are present: a) sample size is justified based on appropriate power calculations (power=80); b) using a multivariate approach 10 cases per IV are used; c) using several correlations or t-tests, a sample of 80 or more reflects adequate power; d) study has sufficient statistical power to detect clinically important effects as statistically significant and record power > 80. Select 'No' if: a) Sample size and power are not reported; b) the above cut-offs are not met.

   Check all that apply.

   - [ ] Yes
   - [ ] No

4. **Was sample drawn from more than one site? ***

   This refers to physical location – multiple groups belonging to the same system count as multi-site. Several units within the same hospital do not count as multi-site, but several hospitals within the same system or region do. Select 'Yes' if the assumptions made above are accomplished. Select 'No' if the assumptions made above are not accomplished, or not reported.

   Check all that apply.

   - [ ] Yes
   - [ ] No

5. **If there were groups in the study, is there a statement that groups are matched in design or statistically adjusted?** *

Select 'Yes, matched in design' if the authors stated clearly that the groups were matched (i.e. gender, unit). Select 'Yes, statistically adjusted' if groups were statistically adjusted for confounder variables (i.e. use of covariance). Select 'Yes, matched in design and statistically adjusted' if the authors clearly report having done both, a) and b). Select 'No, not matched/adjusted' if authors did not state that groups were matched or adjusted. Select 'N/A' if the study included only one group.

Check all that apply.

- [ ] Yes, matched in design
- [ ] Yes, statistically adjusted
- [ ] Yes, matched in design and statistically adjusted
- [ ] No, not matched/adjusted
- [ ] N/A

6. **In case of surveys: was the response rate > 50%?** *

Response rate is operationally defined as the number of people who participated divided by the number of people who were sampled (e.g., given or sent or offered a questionnaire). If not reported, information that allows calculation will be sought and the same rule applied. Select 'Yes' if the response rate is more than 50%. Select 'No' if the response rate is less than 50% or not reported. Select 'N/A' if the study was not a survey.

Check all that apply.

- [ ] Yes
- [ ] No
- [ ] N/A

Measurement

7. **How was (were) the dependent variable(s) measured?** *

Check all that apply.

- [ ] Directly measured (i.e., observed) or taken from an administrative database or chart
- [ ] Self-reported

8. **Did the researchers use instruments with reported reliability and validity (previously or for this study)?** *

Select option 1 if researchers report reliability indices for each research tool they used, and instruments are reliable (intra-rater and/or inter-rater reliability of the outcomes measure was ICC > 0.70 or kappa ≥ 0.70 or at least 80% agreement; Internal consistency [Cronbach's Alpha] for a scale is > 0.70). Select option 2 if the researchers report validity assessments for each research tool they used and the tools are valid (some form of validation was described for the tools used, e.g., face, content, response process, construct, concurrent validity). Select option 3 if the researchers report reliability indices and validity assessments for each research tool they used and tools are reliable and valid. Select option 4 if researchers do not report any reliability indices or validity assessments for the used research tools or tools are not reliable and valid.

Check all that apply.

- [ ] Reliability indices
- [ ] Validity assessments
- [ ] Both, reliability indices and validity assessments
- [ ] No

Statistical Analysis

N/A can only be selected if the respective item is not applicable to the design of the study

9. Was (were) the statistical test (s) used appropriate for the main outcome (i.e., research use)? *

Take into account the assumptions that need to be met for certain statistical tests. For example, a t-test requires continuous, normally distributed variables and is inappropriate when the outcomes are categorical.

Check all that apply.

- [ ] Yes
- [ ] No

10. Were p values reported? *

Select N/A if the study was just descriptive and did not intend to assess any statistical associations

Check all that apply.

- [ ] Yes
- [ ] No
- [ ] N/A

11. Were confidence intervals reported? *

Select N/A if the study was just descriptive and did not intend to assess any statistical associations

Check all that apply.

- [ ] Yes
- [ ] No
- [ ] N/A

12. Were missing data managed appropriately? *

Select 'N/A' if you are certain there are not missing data.

Check all that apply.

- [ ] Yes
- [ ] No
- [ ] N/A
