# Supplementary material for: Effective strategies to motivate nursing home residents in oral care and to prevent or reduce responsive behaviors to oral care: A systematic review
Source: PLoS One. 2017 Jun 13;12(6):e0178913. doi: 10.1371/journal.pone.0178913 (PMC5469468; doi:10.1371/journal.pone.0178913)
Supplement: S4 Appendix — (PDF) [file pone.0178913.s004.pdf]

S3 Appendix: Detailed quality ratings of each included study

Quality (risk of bias) rating of included clinical studies

Study	Selection bias	Study design	Confounders	Blinding	Data collection	Withdrawals and dropouts	Score	Quality rating
Connell et al. (2002) [91]	1	1	1	1	2	3	9/18=0.50	Weak
Jablonski et al. (2011) [65]	1	2	1	1	3	1	9/18=0.50	Weak
Sloane et al. (2012) [92]<br>Zimmerman et al. (2012) [93]<br>Sloane et al. (2013) [66]<br>Zimmerman et al. (2014) [94]	1	2	1	2	1	3	10/18=0.56	Low moderate

Possible scores: 1 = Weak, 2 = Moderate, 3 = Strong
Quality rating: Score ≤ 0.50 = Weak, Score 0.51–0.66 = Low Moderate, Score 0.67–0.79 = High Moderate, Score ≥ 0.80 = Strong

Quality (risk of bias) rating of included cross-sectional studies

Study	Probability sampling used?[a]	Individual representative?[b]	Sample size justified?[a]	Sample drawn from more than one site?[a]	Groups matched in design or statistically adjusted?[c]	Response rate > 50%?[d]	How was (were) dependent variable(s) measured?[e]	Tools used reliable/valid?[f]	Statistical test(s) used appropriate?[d]	*P* values reported?[d]	Confidence intervals reported?[d]	Missing data managed appropriately?[d]	Score	Quality rating
Wilson et al. (2013) [95]	0	0	0	1	1	NA	1	1	0	1	0	NA	5/13=0.38	Weak

Possible scores:
[a]0 = No, 1 = Yes
[b]0 = Not likely, 1 = Somewhat likely, 2 = Very likely
[c]NA = Not applicable, 0 = No, not matched/adjusted, 1 = Yes, matched in design or statistically adjusted, 2 = Yes, matched in design and statistically adjusted
[d]NA = Not applicable, 0 = No, 1 = Yes
[e]0 = Self-reported, 1 = Directly measured
[f]0 = Neither valid nor reliable, 1 = Either reliable or valid, 2 = Both, reliable and valid
Quality rating: Score ≤ 0.50 = Weak, Score 0.51–0.66 = Low moderate, Score 0.67–0.79 = High moderate, Score ≥ 0.80 = Strong
